# The Use of Water in the Treatment of Fever

**Published:** 1857-09

**Authors:** 


*The Use of Water in the Treatment of Fever. By Isaac Casselberry, M. D., of Evansville, Ind.*

This is a pamphlet of 21 pages, reprinted from the *American Journal of Medical Sciences.* The writer occupies the first half of it in considering the functions of the skin, lungs, etc., as a basis for explaining the action of water upon the system, whether used internally or externally. The pamphlet is worthy of a careful perusal.

